# Habitat generalist species constrain the diversity of mimicry rings in heterogeneous habitats

**DOI:** 10.1038/s41598-021-83867-w

**Published:** 2021-03-03

**Authors:** Irina Birskis-Barros, André V. L. Freitas, Paulo R. Guimarães

**Affiliations:** 1 School of Natural Sciences, University of California, Merced, CA 95340 USA; 2 Departamento de Ecologia, Instituto de Biociências, Universidade de São Paulo, São Paulo, SP Brazil; 3 Departamento de Biologia Animal and Museu da Biodiversidade, Instituto de Biologia, Universidade Estadual de Campinas, Campinas, SP Brazil

**Keywords:** Coevolution, Mullerian mimicry, Ecological networks, Evolutionary theory

## Abstract

How evolution creates and maintains trait patterns in species-rich communities is still an unsolved topic in evolutionary ecology. One classical example of community-level pattern is the unexpected coexistence of different mimicry rings, each of which is a group of mimetic species with the same warning signal. The coexistence of different mimicry rings in a community seems paradoxical because selection among unpalatable species should favor convergence to a single warning pattern. We combined mathematical modeling based on network theory and numerical simulations to explore how different types of selection, such as mimetic and environmental selections, and habitat use by mimetic species influence the formation of coexisting rings. We show that when habitat and mimicry are strong sources of selection, the formation of multiple rings takes longer due to conflicting selective pressures. Moreover, habitat generalist species decrease the distinctiveness of different mimicry rings’ patterns and a few habitat generalist species can generate a “small-world effect”, preventing the formation of multiple mimicry rings. These results may explain why the coexistence of mimicry rings is more common in groups of animals that tend towards habitat specialism, such as butterflies.

## Introduction

The evolutionary consequences of species interactions shape trait patterns at the community level and affect the organization of interacting assemblages^1,2^. Phenotypic convergence is an example of community level pattern shaped by species interactions. Examples of phenotypic convergence are the shape of flowers sharing similar pollinators^3^, the chemical composition of fruits consumed by similar vertebrates^4^, and the same warning signals of unpalatable species, i.e., Müllerian mimicry^5^. Understanding under which conditions ecological interactions favor trait convergence or lead to the evolution of trait divergence is still an open question.

Theoretical and empirical evidences demonstrated the role of selection imposed by positive interactions in shaping convergence among species^1,6,7^. In Müllerian mimicry, selection favors convergence because unpalatable individuals from different species with similar warning signals suffer low per-capita predation due to the shared costs of predators learning^8–10^. In fact, similar phenotypic patterns in Müllerian mimicry are mainly due to selection favoring convergence in color patterns^11,12^, although other evolutionary processes, such as shared evolutionary history^13,14^ can produce similar results. In this context, Müllerian mimicry represents a useful study system to better understand under which scenarios selection imposed by mutualistic ecological interactions leads to trait convergence.

**Figure 1:**
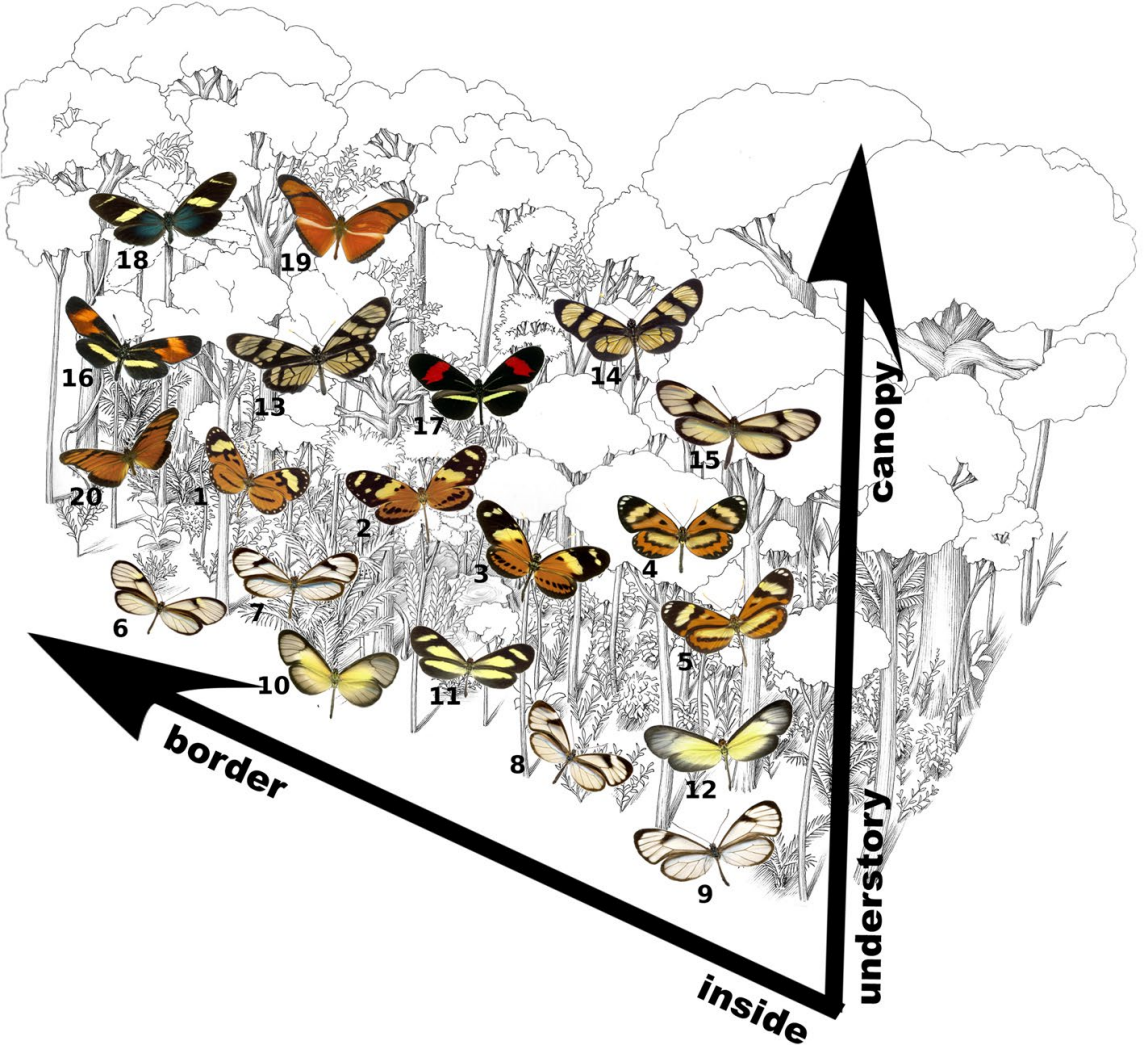
Examples of coexisting mimicry rings in the same local community: tiger (species 1, 2, 3, 4 and 5), blue clearwing (species 6, 7, 8 and 9), yellow clearwing (10, 11 and 12), Methona (13, 14 and 15), erato (16 and 17), sara (18) orange (19 and 20) These butterflies are habitat specialists, using for example a specific part of the forest, such as a certain high of the forest (vertical stratification^37^) or the border of the forest^54^. Species: 1. *Hypothyris euclea laphria*; 2. *Melinaea ludovica parayia*; 3. *Heliconius numata robigus*; 4. *Placidina euryanassa*; 5. *Hypothyris ninonia daeta*; 6. *Episcada striposis*; 7. *Ithomia* *drymo*; 8. *Hypoleria alema proxima*; 9. *Pseudoscada acilla acilla*; 10*. Episcada hemixanthe*; 11. *Aeria olena*; 12. *Scada karschina karschina*; 13. *Lycorea ilione*; 14. *Methona themisto*; 15. *Episcada philoclea*; 16. *Eresia lansdorfi*; 17. *Heliconius erato phyllis*; 18. *Heliconius sara*; 19. *Dryas iulia*; 20. *Dione juno juno*. (Background art by Danilo B. Ribeiro and Rogério Lupo).

Mimicry among defended species has been found in birds^15^, bumblebees^16,17^, butterflies^18^, fishes^19^, frogs^20,21^, and even plants^22^. Whereas in some species-rich communities, multiple defended species converge to the same warning signal^23,24^, in other communities, multiple mimicry rings are known to coexist locally^14,25,26^ (Fig. 1). Empirical and theoretical studies have shown that habitat heterogeneity can explain the coexistence of multiple mimicry rings^27–30^.

**Figure 2:**
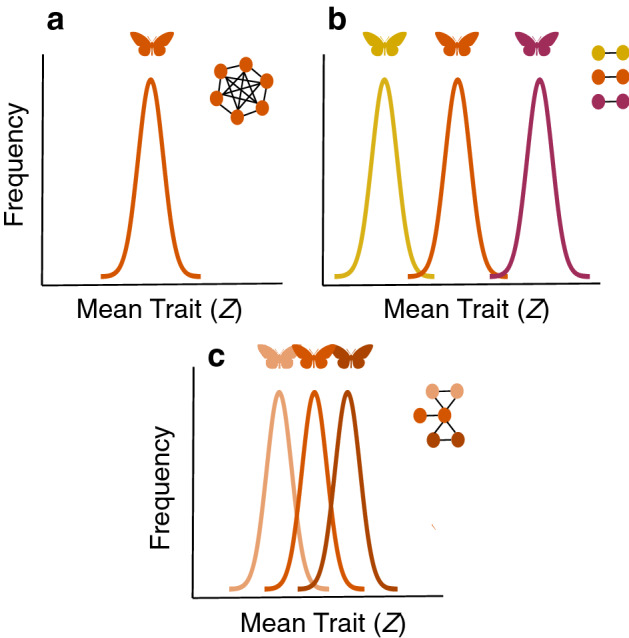
A conceptual example of the expected frequencies of traits values (Z), here representing the color pattern of the butterflies, for distinct scenarios of species specialization in habitats. Circles represent species and species that co-occur are represented by links. **(a)** All species occur in all habitats. In this case only one ring is formed. **(b)** Distinct species occur in different habitats, each species being habitat specialist. In this case all species in each habitat formed a mimicry ring. **(c)** One species is habitat generalist occurring in all habitats—supergeneralist species. In this case each habitat formed a mimicry ring, however their trait values are very similar.

At local scale, species in different habitats may face distinct predators and different environmental selective pressures. The contrast with the background, for example, is essential for the success of aposematic species, since greater contrast allow the consumer to better recognize the signal^31–33^. Different habitats may have different background and, consequently, different selective pressure over the contrast of aposematic species. Additionally, different color patterns can be selected by the habitat for thermal regulation, since different colors have different capacity to absorb solar radiation^34–36^. Nevertheless, the persistence of multiple sympatric mimicry rings may be not only related with habitat heterogeneity, but also how species use the habitat. In fact, Müllerian rings in butterflies are often associated with forest strata^25,37,38^. Willmott and Mallet^39^, for example, showed that host-plant use may constrain the movement of Ithomiini butterflies (Nymphalidae), which helps to maintain mimetic diversity at local scales. In this sense, habitat generalists, by linking otherwise distinct groups of unpalatable species, may prevent the emergence of multiple mimicry rings even if the community is structured in heterogeneous habitats (Fig. 2).

We used a coevolutionary model based on network theory to explore the conditions that favor the emergence of coexisting mimicry rings. In the past years, the use of mathematical models of discrete traits and frequency dependent selection improved our understanding on the evolution of Müllerian mimetism^5,28,30^. Here, we use an alternative approach integrating a standard quantitative genetics phenotypic model combined with network and coevolutionary theories, to address three main questions: (1) What are the roles that different sources of selection play in the formation of multiple mimicry rings? (2) How important is habitat-based selection in shaping mimicry ring patterns within a community? (3) How does the presence of generalist species influence the formation of multiple mimicry rings?

## Methods

### Model

Our model was built upon coevolutionary models for trait evolution in ecological networks^40,41^. We modeled the evolution of a single trait, which describes a signal of a given unpalatable species, such as warning colors. We assumed that it is a continuous trait, with multiple genes contributing to its phenotypic expression such that the warning signal can be described as a real number associated with each individual in a given population^42^. By assuming a continuous trait, we are able to explore in greater detail the patterns of convergence without losing generality. We modeled directly the evolution of the mean trait values for a given species *i*, *Z*_*i*_. In our model, mean trait values evolve as a consequence of selection imposed by different factors, and species with similar trait values form mimicry rings. All variables and parameters used in this model are listed in Table 1. Moreover, we developed an analytical approximation in SI to provide insight on the ways conflicting selective pressures shape coevolution in mimicry rings.

**Table 1:** Model variables and parameters and their descriptions.

Variables/parameters	Descriptions
${Z}_{i}$	Mean trait value of species *i*
${h}_{i}^{2}$	Heritability of trait *Z*_*i*_
${\sigma }_{{F}_{i}}^{2}$	Phenotypic variance of trait *Z*_*i*_
$\frac{\partial {ln(W}_{i})}{\partial {Z}_{i}}$	Selection gradient
${\xi }_{i}$	Fitness sensitivity to change
${E}_{i,k}^{t}$	Partial selection differential due to habitat *k*
${M}_{ij}^{t}$	Partial selection differential due to mimetism
${\sigma }_{{G}_{i}}^{2}$	Additive genetic variance
$p$	Strength of habitat selection
${\theta }_{k}$	Trait value favored by habitat *k*
${q}_{ij}$	Strength of mimetic selection
${m}_{ij}$	Trait matching between species *i* and *j*
${a}_{ij}$	Effect of abundances of co-occurring species
${a}_{i}^{(k)}$	Abundance of species *i* at habitat *k*
${A}_{k}$	Abundance of all species at habitat *k*
$\alpha$	Weight of evolutionary effect of trait matching
$S$	Species richness
$N$	Number of habitats
${N}_{i}$	Total number of habitat species *i* occurs

Initially the mean trait value of a given species *i*, *Z*_*i*_, was randomly sampled from a uniform distribution between 0 and 1. Trait evolution was modeled as discrete events, which means we observed how the mean trait (*Z*_*i*_) of a species change from one generation (*t*) to the next (*t* + *1*)*. Z*_*i*_^*t*^ changes due to both selective pressures imposed by the habitats in which the species occurs, and selective pressures imposed by the co-occurrence of other aposematic species *j*. We assumed selection imposed by the habitat favors particular traits, e.g., conspicuous colors when facing a particular habitat background^33^. We defined habitat-based selection as the selection imposed by the environment where the species occurs, including abiotic pressures, but it could be any other selective pressure on the color that is not directly imposed by mimicry, such as sexual selection. We also assumed that aposematic species that co-occur in the same habitat share the same potential predators^29^. As a consequence, selection mediated by the co-occurrence of other aposematic species and due to the presence of a common predator favors convergence in the trait values.

We assumed that the same predator population consumes species within a single habitat (see empirical evidence^43^). Theoretical and empirical studies have also shown that the way predators use the habitat leads to a concordant habitat use by mimetic species, acting as a selective force that favors the convergence of prey niches and their aposematic traits^29,44^. Predator–prey interactions may lead to complex eco-evolutionary feedbacks between traits and abundances of both predator and prey^45^. As a first approximation, we assumed there is no eco-evolutionary feedback by assuming that the predator has a generalist diet and does not rely on the aposematic species in the model for survival. In fact, some predators that attack Müllerian mimics are generalists, such as insectivorous birds^46^. Moreover, generalist predators are likely to impose stronger selection favoring Müllerian mimicry^47^. This set of assumptions allows us to model the trait evolution in aposematic species without explicitly describing predator ecological or evolutionary dynamics.

Selection imposed by habitats and mimetic selection imposed by co-occurring aposematic species through shared predators determine the selection gradient, shaping the evolution of the mean trait value of species *i* at time *t*, *Z*_*i*_^*t*^. The evolution of trait t in the next time step is defined as:

1 $${Z}_{i}^{t+1}={Z}_{i}^{t}+{h}_{i}^{2}{\sigma }_{{F}_{i}}^{2}\frac{\partial {ln(W}_{i})}{\partial {Z}_{i}}$$

in which ${h}_{i}^{2}$ is the heritability of trait *Z*_*i*_, ${\sigma }_{{F}_{i}}^{2}$ is the phenotypic variance of ${Z}_{i}^{t}$ and both ${h}_{i}^{2}$ and ${\sigma }_{{F}_{i}}^{2}$ are assumed to be fixed and not affected by habitat variation. The selection gradient, $\frac{\partial {ln(W}_{i})}{\partial {Z}_{i}}$, describes how changes in the mean trait value, ${Z}_{i}$, affect the mean fitness of the population of species *i*, ${W}_{i}$. We assumed a linear selection gradient:

2 $$\frac{\partial ln({W}_{i})}{\partial {Z}_{i}}={\xi }_{i}\left({E}_{i,k}^{t}+{M}_{ij}^{t}\right)$$

in which ${\xi }_{i}$ is the sensitivity of the adaptive landscape to changes in trait towards the optimum.${E}_{i,k}^{t}$ and ${M}_{ij}^{t}$ are the partial selection differentials generated by habitat selection and mimetic selection (see Supplementary Fig. S1). We assume species are already aposematic (as in Müller’s model^5^) and we are not modeling the evolution of a new phenotype that is rare and unknown for the predator. Using the fact that ${h}_{i}^{2}={\sigma }_{{G}_{i}}^{2}/{\sigma }_{{F}_{i}}^{2}$, in which ${\sigma }_{{G}_{i}}^{2}$ is the additive genetic variance, Eqs. (1) and (2) lead to:

3 $${Z}_{i}^{t+1}={Z}_{i}^{t}+\varphi \left({E}_{i,k}^{t}+{M}_{ij}^{t}\right)$$

in which $\varphi ={\sigma }_{{G}_{i}}^{2}{\xi }_{i}$ is a scaling parameter controlling the rate of directional change in ${Z}_{i}^{t}$ due to the shape of the adaptive landscape and available additive genetic variance. We now describe the habitat contribution to the selection differential as:

4 $${E}_{i,k}^{t}= {d}_{i,k}{p}_{i,k}\left({\theta }_{k}-{Z}_{i}^{t}\right)$$

in which ${d}_{i,k}=1$ if species *i* occurs at habitat *k* and zero otherwise, ${p}_{i,k}$ is the strength of selection imposed by habitat *k* on species *i*, and ${\theta }_{k}$ is the trait value favored by selection imposed by habitat *k* on species *i*. Thus, if species are specialist and occur in the same habitat, they will have the same ${\theta }_{k}$ value. For the sake of simplicity, we assume that all specialists occurring at the habitat *k* will have the same *p* value. We randomly sampled ${\theta }_{k}$ from a uniform distribution between 0 and 1.

We describe the partial selection differential due to mimetism as:

5 $${M}_{ij}^{t}= \sum_{j}^{S}{q}_{ij}\left({Z}_{j}^{t}-{Z}_{i}^{t}\right)$$

*q*_*ij*_ is the strength of mimetic selection of species *j* on species *i*. We assume that selection favors trait similarity between aposematic species, ${Z}_{i}^{t}={Z}_{j}^{t}$ (see Supplementary Fig. S1) and *S* is the total number of species. We define ${\sum }_{j}^{S}{q}_{ij}=1-p$ and the evolutionary effect *q*_*ij*_ is a function of both the abundance and the trait matching of mimetic species and is defined as:

6 $${q}_{ij}=(1-p)\frac{{a}_{ij}{m}_{ij}^{t}}{\sum_{n=1}^{S}{a}_{in}{m}_{in}^{t}}$$

in which ${a}_{ij}$ is the effect of abundances of species *i* and *j* on ${q}_{ij}$, and ${m}_{ij}^{t}$ is the trait matching of species *i* in relation to *j* at each time step *t* (see below). We assumed that ${a}_{ij}$ is a function of the abundances and co-occurrence of species *i* and species j across habitats:

7 $${a}_{ij}=\frac{\sum_{k=1}^{N}\frac{{a}_{i}^{(k)}{a}_{j}^{(k)}}{{A}_{k}}}{\sum_{r=1}^{S}\sum_{n=1}^{N}\frac{{a}_{i}^{(n)}{a}_{r}^{(n)}}{{A}_{n}}}$$

in which ${a}_{i}^{(k)}$ is the abundance of species *i* at habitat *k*, ${a}_{j}^{(k)}$ is the abundance of species *j* at habitat *k*, ${A}_{k}$ is the abundance of all *S* unpalatable species at habitat *k*, and *N* is the total number of habitats. The species abundances in the habitat *k,* ${a}_{i}^{(k)}$ is an integer number randomly sampled from a uniform distribution between 1 and 10, representing the comparative abundance between species and they are fixed during the simulations. We used fixed abundances as a simplifying assumption because (1) unpalatable *Heliconius* butterflies show populations that appear to be at steady state over time^48,49^, and (2) Müllerian mimicry is not expected to generate eco-evolutionary feedbacks predicted to occur in Batesian mimicry, in which the mimic species are palatable^50^. Said that, we performed a sensitivity analysis to explore the effects of species abundances in the evolutionary dynamics (Supplementary Fig. S2).

We defined trait matching as

8 $${m}_{ij}^{t}=\frac{{e}^{{-\alpha \left({Z}_{j}^{t}-{Z}_{i}^{t}\right)}^{2}}}{{\sum }_{l=1}^{S}{b}_{il}\left[{e}^{{-\alpha \left({Z}_{l}^{t}-{Z}_{i}^{t}\right)}^{2}}\right]}$$

where $\alpha$ weights the evolutionary effect of trait matching between species and ${b}_{il}=1$ if both species co-occur at least in one habitat (${\sum }_{k}{a}_{i}^{(k)}{a}_{j}^{(k)}$), and ${b}_{il}=0$ otherwise. Hence, ${m}_{ij}^{t}$ describes the evolutionary effects of a species on the adaptive landscape of another species, describing therefore the effects of trait matching between co-mimic species, whereas ${a}_{ij}$ describes the density-mediated (i.e., abundances and number of co-occurring habitats) of the pair-wise co-occurrence between co-mimic species. Although equation that describes how the trait change is linear (Eq. 2)—a usual approach in quantitative genetics—Eq. (8) shows a non-linear dependence of selection gradient on trait matching in our model (Supplementary Fig. S3). This non-linearity of trait matching effects might be also amplified by the effects of species abundance in the model.

Because speciation events are unlikely to occur in a short period of time, we assumed that species richness, *S*, is fixed. Also, we simulated our model in a species rich-community because Müllerian mimicry rings are more likely to be formed in species-rich communities^47^*.* We also assumed that our populations are large enough that genetic drift has a negligible effect. We considered the formation of a mimicry ring when the mean trait ${Z}_{i}$ of different species converge to similar trait values. We explored the effects imposed by local selective pressure and species habitat generalists using distinctive parameterizations (scenarios) of the model. For all scenarios, we simulated the model over 1000 times step, which was long enough to permit examination of steady state conditions, and fixed parameter values were: number of species *S* = 50, number of habitat *N* = 10, scaling parameter *φ* = 0.25, and weight of evolutionary effect between species $\alpha =2$. Other values of these parameters do not qualitatively change the results (Supplementary Figs. S4-S7). All simulations were run in R 3.1.3^51^.

### How do sources of selection and habitat heterogeneity influence the formation of mimicry rings?

**Figure 3:**
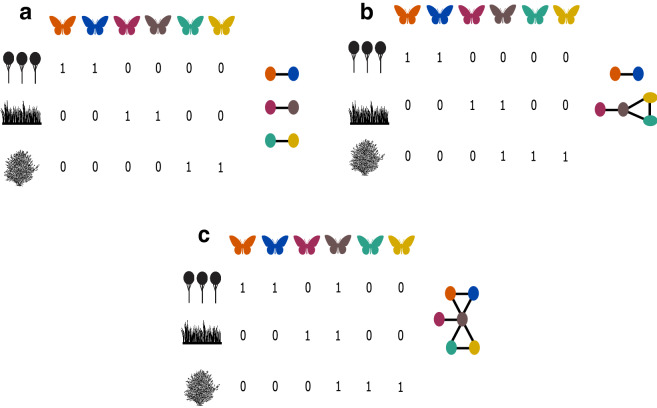
We use co-occurrence matrices (and their network representations) to explore the influence of habitat generalist species in the evolution of mimicry rings. In these matrices each column represents a distinct aposematic species and each line represent a distinct habitat. **(a)** *Perfectly modular scenario*—where each species only occurs in only one habitat. (**b**) In this scenario the brown species occurs in more than one habitat, connecting species purple with species green and yellow. (**c**) In this scenario brown species is a supergeneralist—occurring in all habitats and connecting all species.

To explore the effect of habitat heterogeneity in the formation of mimicry rings we set ${\theta }_{i,k}={\theta }_{k}$ for any species *i* and habitat *k*, i.e., each habitat has a different optimum value and this optimum value was identical for all species occurring in that habitat (${\theta }_{k}$). We also assume each habitat has a distinct assemblage of species (i.e., species are habitat specialists—*perfectly modular scenario—*Figs. 2b and 3a). Thus, the community is heterogeneous by having distinct habitats with different optima. In our simulations, the community is structured in 10 different habitats and each habitat has five habitat specialist species. We then used three different approaches to explore the role of different sources of selection and habitat heterogeneity in the formation of mimicry rings. First, both habitat and mimetic selection affect trait evolution (baseline scenario, *p* = *0.3*). Second, we analyzed the sole effect of habitat heterogeneity on trait evolution by removing the effect of mimics (*p* = *1*). Third, we assumed that there was no habitat-based selection (*p* = *0*).

To gain a better understanding of the importance of habitat-based selection on the formation of mimicry rings, we also explored how different habitat-based selection strength affects coevolutionary dynamics. We used a set of simulations varying values of *p* from 0 to 1 by 0.1. For each value of *p* we ran 100 simulations. In order to explore the distinctness of mimicry rings we analyzed the variance of species trait value (*Z*_*i*_) among species after 1000 times steps in our simulation.

### How does the presence of habitat generalist species influence the formation of mimicry rings?

Starting with a *perfectly modular scenario,* we randomly sampled one species from our community to occur in all habitats, thus simulating a supergeneralist (SG) species (Figs. 2c and 3c). By doing that we are assuming that different species have different habitat tolerances. We also assumed that dispersal of species between habitats is implicit by the co-occurrence of species (Fig. 3). Because the supergeneralist species *i* occurs in multiple habitats, the trait value favored by habitat-based selection on the supergeneralist *i* is an average of the trait values favored by selection in the distinct habitats in which species *i* occurs:

9 $${\theta }_{i}=\frac{{\sum }_{k=1}^{N}{{g}_{ik}\theta }_{k}}{{N}_{i}}$$

in which ${g}_{ik}=1$ if the species *i* occurs at habitat *k* and it is zero otherwise, and *N*_*i*_ is the total number of habitat species *i* occurs. We are assuming that supergeneralists use all habitats equivalently, which is a simplifying assumption. To investigate if our results are robust to this assumption, we performed sensitivity analyses in which we (1) calculate the habitat optima of supergeneralists as a weighted average, simulating a preference in one habitat (Supplementary Fig. S8); (2) decreased the value of $\varphi$ for generalists, decreasing the slope in the adaptive landscape for the supergeneralists (Supplementary Fig. S9), implying that even phenotypes far from the mean optimum value would be viable. These sensitivity analyses showed no qualitative change in our results.

Up this point we had just analyzed the effect of one supergeneralist in the community. Next, we varied the number of supergeneralists in our community from five species (10% of species in the community) up to 35 species (70% of species in the community) in increments of 5. We run 100 simulations for each scenario.

Supergeneralists have two distinct effects over the community in our simulations. First, supergeneralists increase the mean number of habitat occurrences per species in the community. For example, in our simulations when one species is a supergeneralist the number of occurrences in the community increases from 50 to 59 (see Fig. 3a,c for an illustration with fewer species). Second, supergeneralists connect otherwise previously isolated habitats, which might lead to qualitatively distinct evolutionary dynamics. We explored if the effects of supergeneralists were a consequence of the increased mean number of habitat occurrences per species by contrasting (1) simulations with supergeneralists in the community with (2) simulations in which the levels of habitat generalization were increased by randomly allowing species to occur in more than one habitat (for a similar approach see^6^). Note, that it is only possible to have connections among rings if we create overleaps among habitats. In this sense, by only adding specialist species in the community without creating connection among habitats it does not change the evolutionary dynamics (Supplementary Fig. S4). We investigated if the variance of species trait value (*Z*_*i*_) among species after 1000 times steps differs between these two scenarios. Departures between scenarios would indicate that the effect of supergeneralists on evolutionary dynamics is not only a consequence of increasing habitat occupation but also a result of supergeneralists directly connecting otherwise isolated set of habitats.

## Results

### How do sources of selection and habitat heterogeneity influence the formation of mimicry rings?

**Figure 4:**
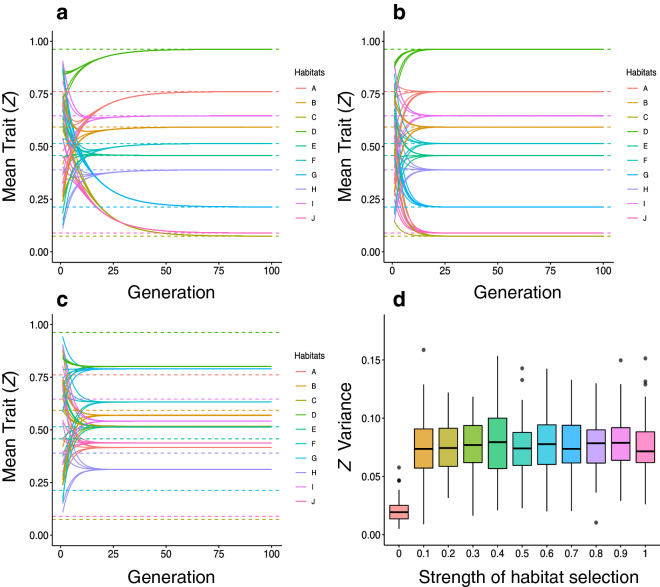
Exploring evolutionary change in trait mean values under mimetic and habitat-based selection. In these simulations species are loyal to their habit, occurring in only one habitat—*perfectly modular scenario*. In (**a-c**) each habitat is represented by a different color, the dashed line is the habitat optima, each continuous line is one species and their color are the same as the habitat optima where they occur. (**a**) The change in trait value in 100 generations when there is both mimetic and habitat-based selection (*p* = 0.3). (**b**) The change in trait value in 100 generations when there is only habitat-based selection (*p* = *1*) and no mimetic selection. (**c**) The change in trait value in 100 generations when there is only mimetic selection occurs and no habitat-based selection (*p* = *0*). **(d)** Variability of trait values after 100 generations in 100 simulation for different strengths of habitat selection.

When all species are habitat specialists, species form mimicry rings in which co-occurring species show the same trait value favored by habitat selection (Fig. 4a). We then used two sets of simulations to explore the role of habitat-based selection and mimetic selection. As expected, when there was just habitat-based selection and no mimetic selection, species converged to habitat optima (Fig. 4b). Similarly, when there was just mimetic selection, distinct mimicry rings were formed (Fig. 4c). This result indicates that habitat-based selection was not essential for the formation of habitat-specific mimicry rings. The interplay between habitat-based and mimetic selection affected coevolutionary dynamics in unexpected ways. If habitat-based and mimetic selective pressures were both affecting trait evolution the time to equilibrium was almost four times as longer than in scenarios in which just one selective pressure was driving trait evolution (Fig. 4a–c and Supplementary Fig. S10).

Sensitivity analysis and an analytical approximation of our model showed that the trait values defining the mimicry rings were not affected by the strength of habitat-based selection when p ≠ 0 (Fig. 4d, SI). Trait evolution led traits to the habitat optima whenever the strength of habitat-based selection was greater than zero (Fig. 4a–c). In this sense, scenarios with a greater variance of habitat optima lead to a greater variance among mimicry rings (Supplementary Fig. S11). In our baseline simulations, we sample the values of habitat optima from a uniform distribution, thus we start our simulation with a scenario that does not favor the formation of groups of habitats with similar thetas. Even though, our results show the formation of different mimicry rings. We also analyzed a scenario where the values of thetas are grouped, favoring the formation of groups and lowering the variance among mimicry rings (Supplementary Fig. S12). If there was no habitat-based selection (*p* = 0), trait values of species from the same habitat approached each other (Fig. 4d). By reducing trait variance within sites and because all species came from the same pool (i.e., were sampled from the same trait distribution), *p* = *0* led to decrease trait variance across distinct habitats and, consequently, the distinctiveness of the mimicry rings (Fig. 4c,d). Thus, habitat selection is not essential to the emergence of mimetic rings but favor the distinctness among different mimetic rings coexisting at the same community.

### How does the presence of habitat generalists influence the formation of mimicry rings?

**Figure 5:**
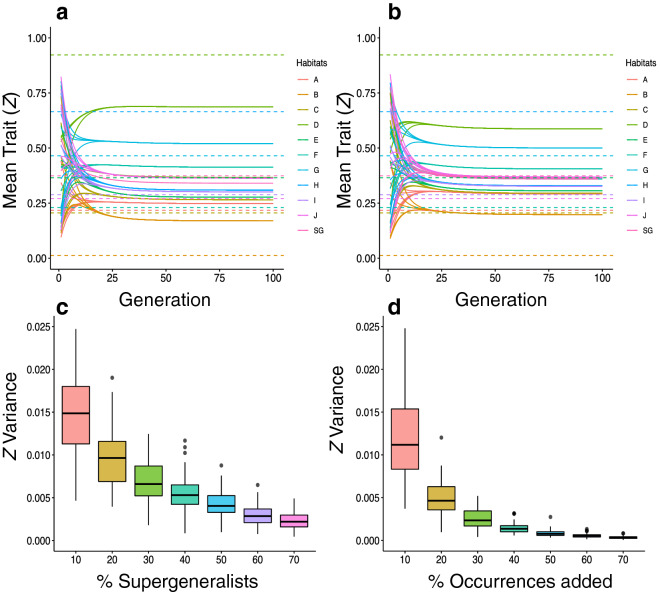
Including supergeneralist, which is a species that occur in all habitats, or occurrences, starting from the *perfectly modular scenario*. In (**a**) and (**b**), each habitat is represented by one color, dashed line is the habitat optima, each continuous line is one species and their color is the same as the habitat optima where they occur. **(a)** The change of trait value in 100 generations when there is only one supergeneralist (SG) in a community. **(b)** The change of trait value in 100 generations when 10% of the species in a community are supergeneralist (SG). **(c)** Variability of trait values after 100 generations in 100 simulations with different percentages of supergeneralist in a community. **(d)** Variability of trait value after 100 generations in 100 simulations with different percentages of occurrences in a community.

Habitat supergeneralists had a major impact on the evolutionary dynamics of mimicry rings. Even a single supergeneralist changed the evolutionary dynamics in two distinct ways. First, the presence of a supergeneralist drove habitat specialists to reach trait values that were not the ones favored by habitat-based selection (Fig. 5a). Second, the presence of supergeneralists decreased the distinctiveness among mimicry rings in the community (Fig. 5b,c, Supplementary Fig. S13). If 10% of species were supergeneralists, the overall trait similarity increased, blurring the differences among mimicry rings (Fig. 5b). The higher the number of habitat supergeneralist*s*, the lower the mimicry ring distinctiveness (Fig. 5c). Ancillary simulations revealed that the effects of supergeneralism in driving convergence in traits of unpalatable species at the community level are faster in the absence of habitat-based selection (Supplementary Figs. S14 and S15).

We then investigated the two effects of habitat supergeneralists on evolutionary dynamics: (1) the presence of supergeneralists, and (2) the increase in the overall level of habitat generalization. When we randomly allowed species to occur in more than one habitat, we obtained smaller trait variability among species than when it was a single supergeneralist (Fig. 5c,d). Thus, increasing the mean level of habitat generalization has a greater influence in trait value evolution than increasing the number of supergeneralists.

We performed additional analyses to explore the drivers of different levels of trait variation in these two scenarios. We tested whether final trait variation was correlated with variation in mean species habitat optima and, because patterns of co-occurrence can create pathways favoring convergence^6^, we tested whether final trait variation was also associated with the path length connecting species. We measured path length as direct and indirect links, in a spatial network in which nodes represent species and links representing that those two species co-occurred in at least one habitat (see Fig. 3). Thus, co-occurring species are directly linked, whereas species that do not co-occur might be indirectly linked by generalists. Final trait variation is a function of variation in mean species habitat optima. In simulations where we increased the overall level of generalism, variations in mean species habitat optima ($\langle {\theta }_{i}\rangle$) and path length were smaller than in simulations where supergeneralists were added to the community, favoring low trait variation among species (Supplementary Figs. S16–S18).

## Discussion

In this study we gave a step forward in the understanding of the coexistence of multiple mimicry rings. We branched the evolutionary dynamics in habitat-based selection and mimetic selection to better understand how these two forces contribute to the formation of mimicry rings. Additionally, we investigated how the use of habitat affect the formation of multiple rings and the role that generalist species play in the evolutionary dynamic. Broadly, this study improves our understanding on how ecological interactions shape trait evolution and, especially, the convergence of traits.

First, we showed that if all species are specialists they evolve towards the habitat optima where they occur. In this way, in a heterogeneous community, specialists in different habitats will diverge in their traits, leading to the formation of distinct mimicry rings as a side-effect of habitat-based selection. On the other hand, if habitats are more homogeneous, selection imposed by habitat may favor similar warning signals, leading to similar color patterns. In fact, the formation of multiple mimicry rings is more common in heterogeneous tropical zones than in homogeneous temperate zones^14,52,53^. As mimicry rings are in part a consequence of habitat-based selection, we should expect that changes in habitat’s features would lead to changes in phenotypes favored by such habitats, resulting in species that converge to different phenotypic patterns. In fact, there is evidence that the composition of mimicry rings does change with habitat disturbance^54^. Although the primary mechanism for these changes could simply result from species sorting, evolutionary dynamics driven by habitat-based selection may also fuel new types of mimicry rings, which in turn may explain why same species are part of distinct mimicry rings across large geographical areas.

It is important to notice that in our model we assumed a generalist predator to avoid the co-evolutionary dynamics between predator and prey. We also considered that predator population only attacks species within a habitat. As a next step it is possible to look at a smaller spatial scale, for example, focusing on species polymorphisms^53,55^. In fact, the emergence of a polymorphism is not only determined by the introgression^56^, but also might be a result of a geographical mosaic selection created by the heterogeneity of predator behavior across space and time^29,30^. For example, different predator foraging at different guilds can lead to polymorphic pattern in prey population^57^. In this sense, future studies should focus on the role of broad intra-specific habitat use of both, predators and prey, to promote the emergence of polymorphism on mimetic species.

Second, our analyses showed that if the unpalatable species are specialized in the same habitat, the selective pressure exerted by the co-occurrence of species is enough for the emergence of distinct mimicry rings in a community. Mutualistic interactions between co-occurring species are known to lead to convergence across multiple ecological traits^1,29^. The degree of convergence may be counterbalanced by interspecific competition, but if selection favoring convergence outweighs the effects of competition, as implicitly assumed in our model, than convergent patterns should emerge^1^. Thus, in this scenario, coevolution would serve as the evolutionary process behind the formation of mimicry rings, supporting the notion that mutualistic interactions have the potential to affect the evolutionary dynamics in diverse communities^58,59^. Our results indicate that the presence of mimicry rings is not indicative of either habitat or mimetic selective pressures shaping warning signals in unpalatable species because either habitat-based selection alone, mimetic selection alone, or both operating in concert, can lead to the formation of mimicry rings. In this sense, although it is more likely that both habitat and mimetic selections are affecting the evolution of Müllerian mimicry^60–62^, we need studies that partition out the importance of each of these components on the trait evolution.

For example, color pattern, one of the most conspicuous composites of traits involved in Müllerian mimicry, perform other functions related to both mimetic and habitat-based selection, such as sexual selection or thermoregulation^35,63,64^. Moreover, color patterns vary in their conspicuousness depending on habitat background, as demonstrated by a variety of organisms including preys^31^, and plant structures^33^, thus indicating that selection imposed by Müllerian mimicry is often habitat-dependent. Therefore, the scenario where multiple selective pressures are acting in traits associated with mimicry is expected, which makes both advergence across habitats (due to habitat-based selection) and convergence due to habitat co-occurrence and coevolution the central processes in the formation of mimicry rings. In this way, our results shed light on the consequences of the complex interplay between different sources of selection. Our results reveal that the time required for species to reach stable trait values slows down when both habitat and mimetic selection are shaping traits due to conflicting selective pressures. Conflicting selection, in addition to stabilizing selection and “the Red King effect”^65^, is one of the multiple processes that slow the pace of evolution in mutualisms^66–69^. However, even the slowest evolutionary change in our simulations occur in a lower number of generations, representing that evolutionary changes can occur in an ecological time scales for short-lived species, such as butterflies.

Our analysis also showed that when there is habitat-based selection, species’ trait is always pulled towards the habitat optimum, in spite of the influence of the abundance effect of the mimicries. This was an unexpected result, since most models shows that the most abundant species serves as the central force for Müllerian mimicry. However, the theoretical prediction that abundances affect the pattern of mimicry emerges from two assumptions: (1) the strength of selection is positively associated with the frequency of interactions—which is an assumption that does not necessarily hold for ecological interactions^70^ and (2) traits are not under local habitat-based selection. In this sense, our model shows that when all species are habitat specialists, they converge to the habitat optima, regardless species abundance. However, when there is a generalist species in the community, species that co-occur in the same habitat no longer reach the habitat optima. In this sense, the presence of a habitat generalist changes their adaptive peaks to a position far from their habitat optima. In the simulations in our studies in which there was no habitat selection, abundance operates as the central force influencing final trait values, with species converging to the trait value of the most abundant species.

Third, our results showed that just a few habitat supergeneralists are required to drive the convergence of all potentially distinct mimicry rings to a single mimicry ring. Our analysis revealed that having more habitat generalists led to faster trait convergence than adding supergeneralists, suggesting that the effects of habitat generalization in collapsing mimicry rings is a consequence of increasing the mean level of generality in the community. Patterns of convergence in species-rich mutualisms were associated with a type of network-based pattern called the “small-world effect”^6,71^, that occurs when there is a short pathway connecting species (nodes) in a community (network)^71,72^. In fact, by adding habitat supergeneralists or increasing the mean level of habitat generalization in the community, we reduced the length of pathways connecting species through patterns of co-occurrence, leading to a rapid convergence in color traits. In a broader perspective, these results illustrate how network theory may provide new insights to coevolutionary dynamics of species interactions^6,73^.

Because even a few supergeneralists or moderate levels of habitat generalization would collapse multiple mimicry rings to a single one, our simulations provide insights on the mechanisms that allow the coexistence of mimicry rings. For instance, *Heliconius* butterflies (Nymphalidae: Heliconiinae) are known to be very loyal to their home-range, roosting and reproductive sites and Ithomiini butterflies (Nymphalidae: Danainae) to habitat type and flight height (vertical stratification)^37,38,74,75^. Furthermore, butterfly mimicry rings are often associated with particular habitats, indicating that the co-occurrence of specialists is congruent with the patterns of mimicry rings. However, in other mimicry groups with broad habitat use, *e.g.* frogs^76^, snakes^77^, velvet ants^78^, the formation of mimicry rings seems indeed to have a large spatial structure, and different mimicry rings are often allopatric. In this sense, our study provides a theoretical concept that could be empirically tested in the future, such as if co-existence of mimicry rings is promoted by the prevalence of habitat-specialist species.

## Supplementary Information

Supplementary Information.
