# Supplementary material for: Habitat generalist species constrain the diversity of mimicry rings in heterogeneous habitats
Source: Sci Rep. 2021 Mar 3;11:5072. doi: 10.1038/s41598-021-83867-w (PMC7930205; doi:10.1038/s41598-021-83867-w)
Supplement: Supplementary file 1 — Supplementary Information. [file 41598_2021_83867_MOESM1_ESM.pdf]

Habitat generalist species constrain the diversity of mimicry rings in heterogeneous habitats

Irina Birskis-Barros[1,2], André V. L. Freitas[3], Paulo R. Guimarães Jr.[2*]

SUPPLEMENTARY MATERIALS

Contents:

1. Analytical approximation 1

2. Figures... 3

1. Analytical approximation

We performed an analytical approximation of the model to explore in which conditions evolutionary dynamics will favor particular phenotypes in a given set of aposematic species. In this analytical approximation we assumed q_{ij} is fixed and we used equations (3) and (4) to generate the equation that describes trait change in a time step:

$$Z_i^{t+1} = Z_i^t + \varphi_i\left(p_i\langle\theta\rangle + \sum_j^S q_{ij}Z_j^t - Z_i^t\right) \quad (S1)$$

The simulations show that traits reach to stationary values after transient coevolutionary dynamics. We investigated the factors affecting trait values at the fixed point following the approach of described at [68]. The trait value fixed point is defined as:

$$Z_i^* = Z_i^* + \varphi_i\left(p_i\langle\theta\rangle + \sum_j^S q_{ij}Z_j^* - Z_i^*\right) \quad (S2)$$

$$Z_i^* = p_i\langle\theta\rangle + \sum_j^S q_{ij}Z_j^* \quad (S3)$$

In matrix form:

$$\mathbf{Z} = \mathbf{P}\Theta + \mathbf{QZ} \quad (S4)$$

$$\mathbf{Z} = (\mathbf{I} - \mathbf{Q})^{-1}\mathbf{P}\Theta \quad (S5)$$

If the species coexist in a single habitat k, then $\langle\theta\rangle = \theta_k$ for all species in the community. As a consequence, S5 implies that all species will converge to θ_k if $p_i > 0$, i.e.,

no matter the strength of habitat-based selection. If there is no habitat-based selection and assuming $\varphi_i = \varphi$ to any species *i* the equation S1 simplifies to:

$$Z_i^{t+1} = Z_i^t + \varphi\left(\sum_j^S q_{ij} Z_j^t - Z_i^t\right) \quad \text{(S6)}$$

In this model, the fixed point is $Z_j^* = Z_i^*$ for all *i* and *j*. The trait value defining the fixed point is dependent of initial conditions and the distribution of values for pair-wise effects between species, q_{ij}. Under the assumption of that $q_{ij} = (1-p)/k_i$, in which k_i is the number of co-mimetic species of *i* in the entire community, for any pair of species *i* and *j* and assuming that for any species *i*, $Z_i^0 = \langle Z \rangle + \varepsilon_i^0$, in which $\langle Z \rangle$ is the mean trait value at the initial time step, then:

$$\langle Z \rangle + \varepsilon_i^1 = \langle Z \rangle + \varepsilon_i^0 + \varphi\left[(1-p)\langle Z \rangle - \left(\langle Z \rangle + \varepsilon_i^0\right)\right] \quad \text{(S7)}$$

Since *p*=0:

$$\langle Z \rangle + \varepsilon_i^1 = \langle Z \rangle + \varepsilon_i^0 - \varphi\varepsilon_i^0 \quad \text{(S8)}$$

$$\varepsilon_i^1 = (1-\varphi)\varepsilon_i^0 \quad \text{(S9)}$$

As a consequence for large time step *t*

$$\varepsilon_i^t = (1-\varphi)^t \varepsilon_i^0 \quad \text{(S10)}$$

$$\lim_{t\to\infty} \varepsilon_i^t = 0 \quad \text{(S11)}$$

Thus, in this approximation $Z_i^* = \langle Z \rangle$ for any species *i,* indicating that, in the absence of environmental selection and assuming similar evolutionary effects across pairs of species, traits converge to the mean initial trait value.

2. Figures

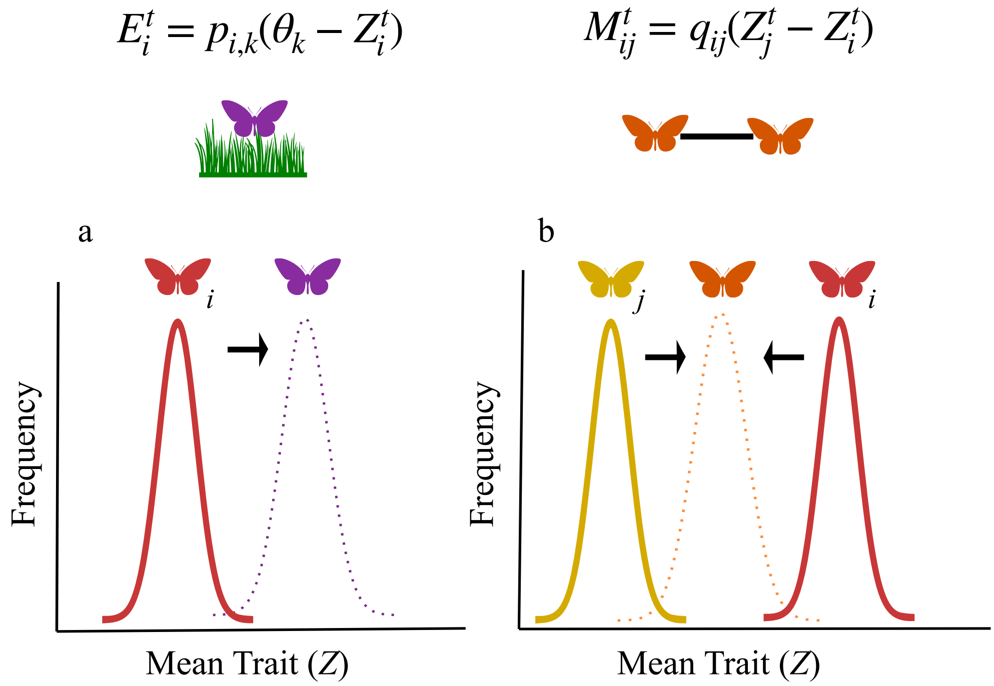

Figure S. 1: A conceptual figure to help the understanding of our model. We are representing the trait optimum favored by **a)** habitat selection. Habitat optimum is $\theta_k$in our model and here is represented by the purple color. **b)** mimetic selection. In this case the selection favors trait matching among species that co-occur. Here this trait matching is represented by the orange color.

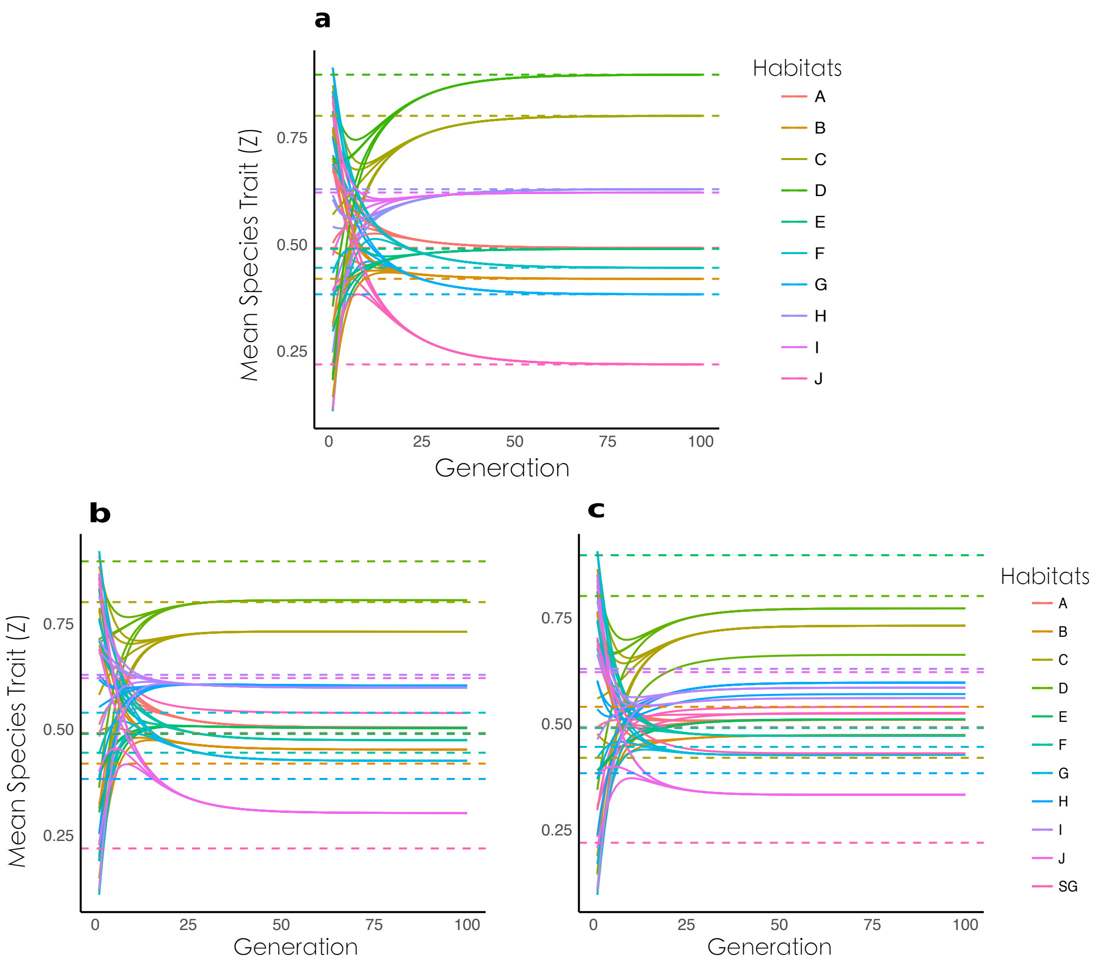

Figure S. 2: Exploring mimetic and habitat-based selection when all species have the same abundance. Each habitat is represented by one color, dashed line is the habitat optima, each continuous line is one species and their color is the same as the habitat optima where they occur. **a)** the change of trait value in 100 generations when all species are habitat specialists, occurring in only one habitat, in a *perfectly modular scenario* **b)** the change of trait value in 100 generations when there is only one supergeneralist (SG) in a community **c)** the change of trait value in 100 generations when 10% of the species in a community are supergeneralist (SG)

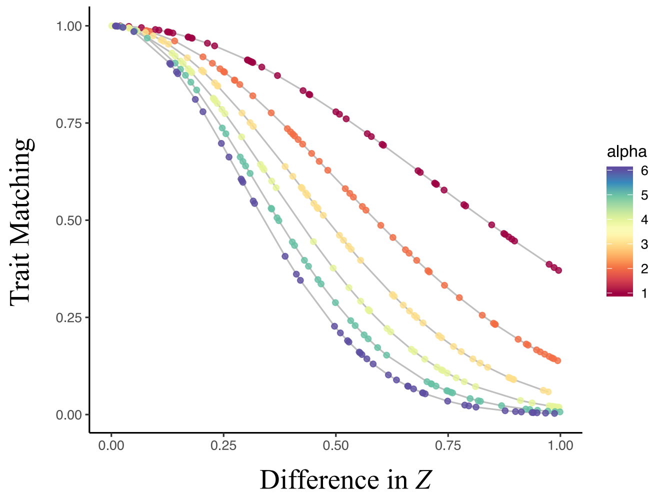

Figure S. 3: Relationship between the difference in the mean trait value among pairs of co-mimetic species and the consequences of trait matching to the selection gradient assuming different values of alpha (different colors). See equation 8 of the main text for additional information.

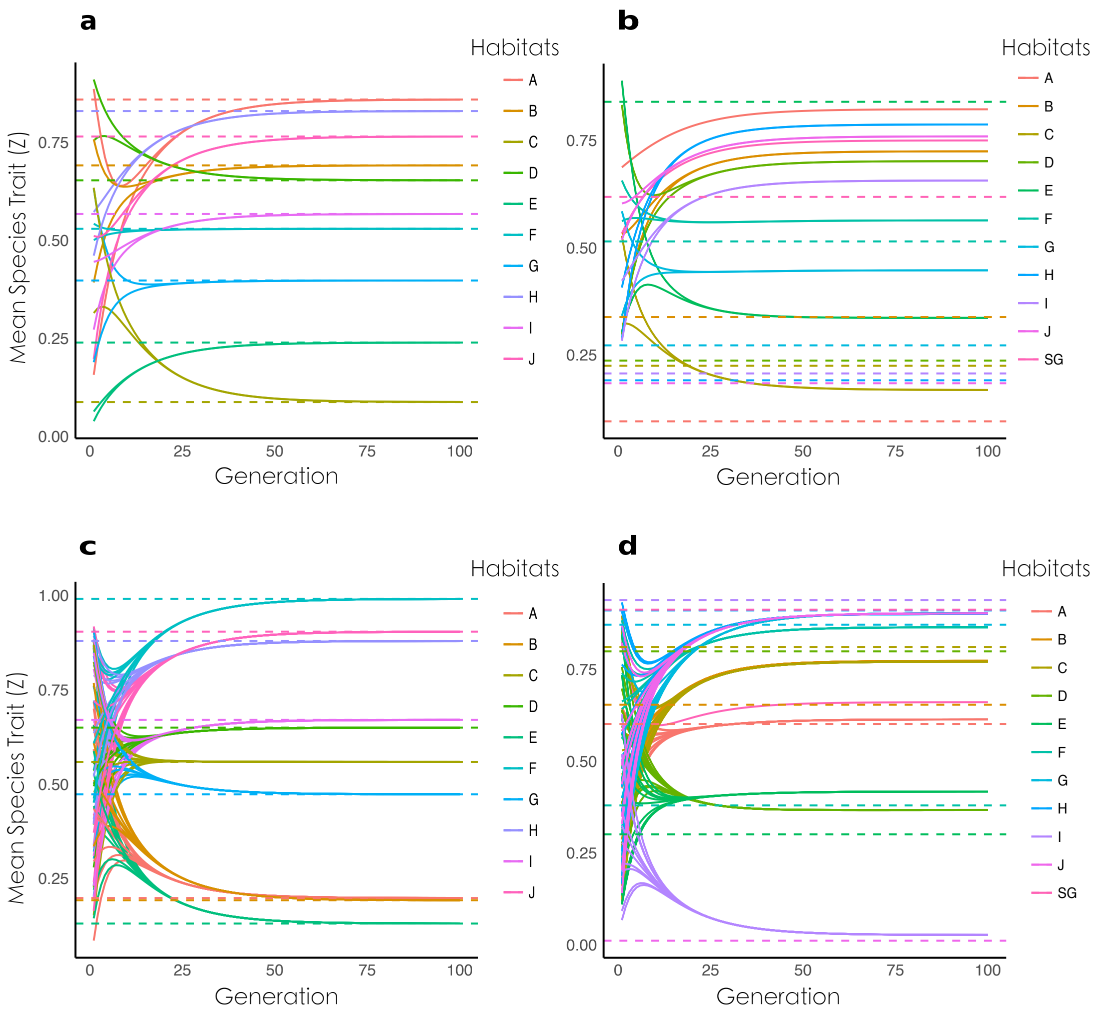

Figure S. 4: Sensitivity analyses of species richness (S). Exploring evolutionary change in trait mean values under mimetic and habitat-based selection. Each habitat is represented by one color, dashed line is the habitat optima, each continuous line is one species and their color is the same as the habitat optima where they occur. In **a-b** species richness (S) is equal to 20 **a)** species are loyal to their habit, occurring in only one habitat, in a *perfectly modular scenario* **b)** one species in the community is a supergeneralist. In **c-d** species richness (S) is equal to 100 **c)** species are loyal to their habit, occurring in only one habitat, in a *perfectly modular scenario* **d)** one species in the community is a supergeneralist.

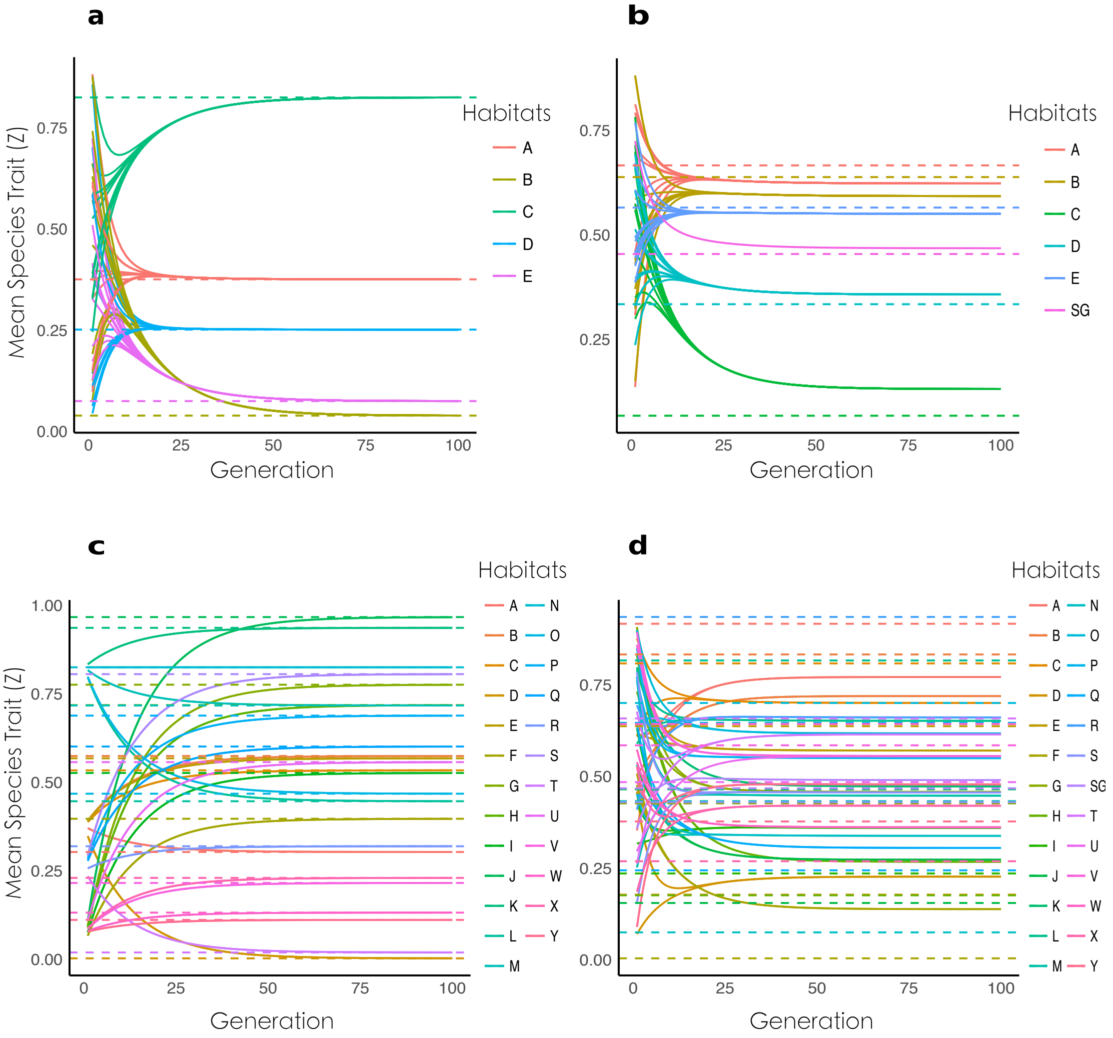

Figure S. 5: Sensitivity analyses of the number of habitats (N). Exploring evolutionary change in trait mean values under mimetic and habitat-based selection. Each habitat is represented by one color, dashed line is the habitat optima, each continuous line is one species and their color is the same as the habitat optima where they occur. In **a-b** the number of habitat (N) is equal to five **a)** species are loyal to their habit, occurring in only one habitat, in a *perfectly modular scenario* **b)** one species in the community is a supergeneralist. In **c-d** the number of habitat (N) is equal to 25 **c)** species are loyal to their habit, occurring in only one habitat, in a *perfectly modular scenario* **d)** one species in the community is a supergeneralist.

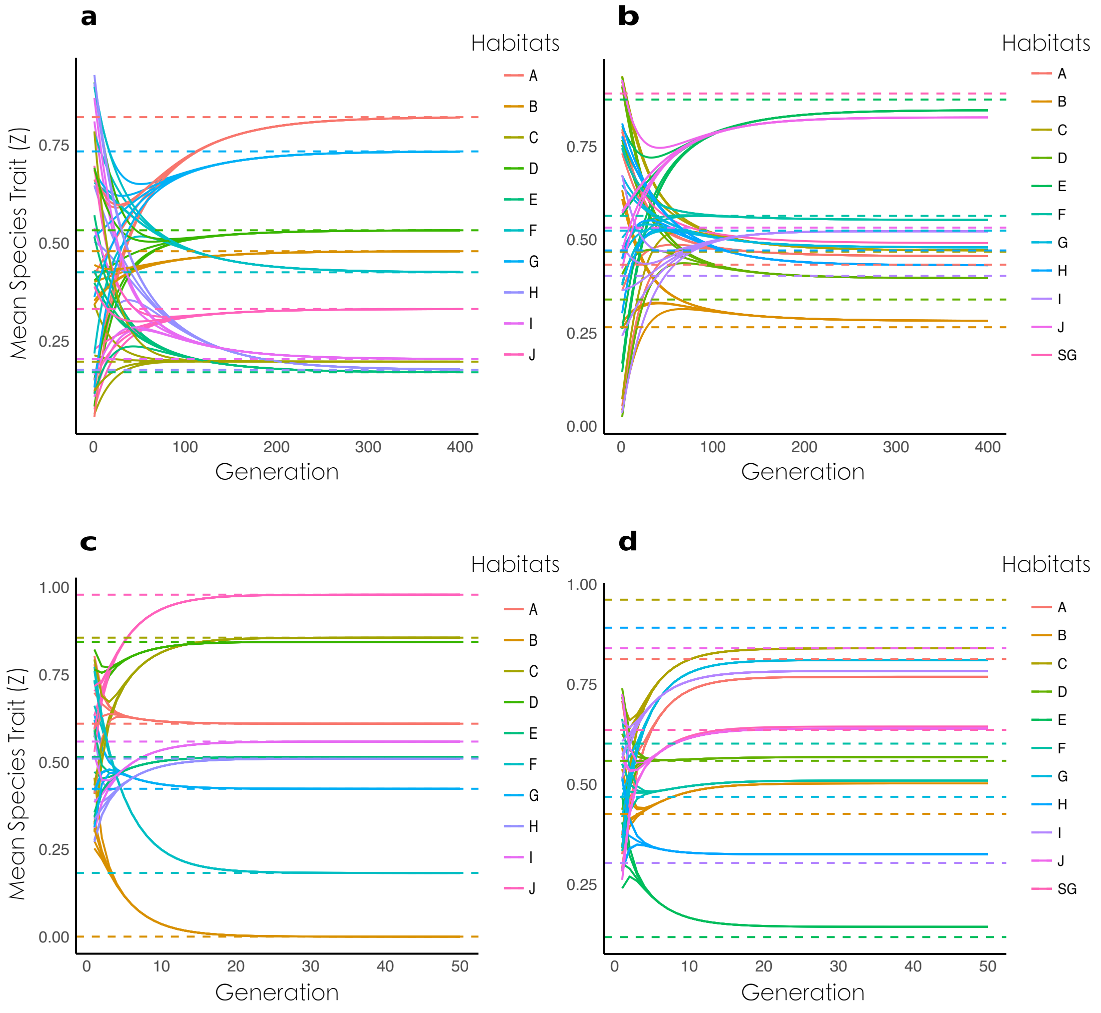

Figure S. 6: Sensitivity analyses of the scaling parameter φ. Exploring evolutionary change in trait mean values under mimetic and habitat-based selection. Each habitat is represented by one color, dashed line is the habitat optima, each continuous line is one species and their color is the same as the habitat optima where they occur. In **a-b** φ is equal to 0.05 **a)** species are loyal to their habit, occurring in only one habitat, in a *perfectly modular scenario* **b)** one species in the community is a supergeneralist. In **c-d** φ is equal to 0.7 **c)** species are loyal to their habit, occurring in only one habitat, in a *perfectly modular scenario* **d)** one species in the community is a supergeneralist.

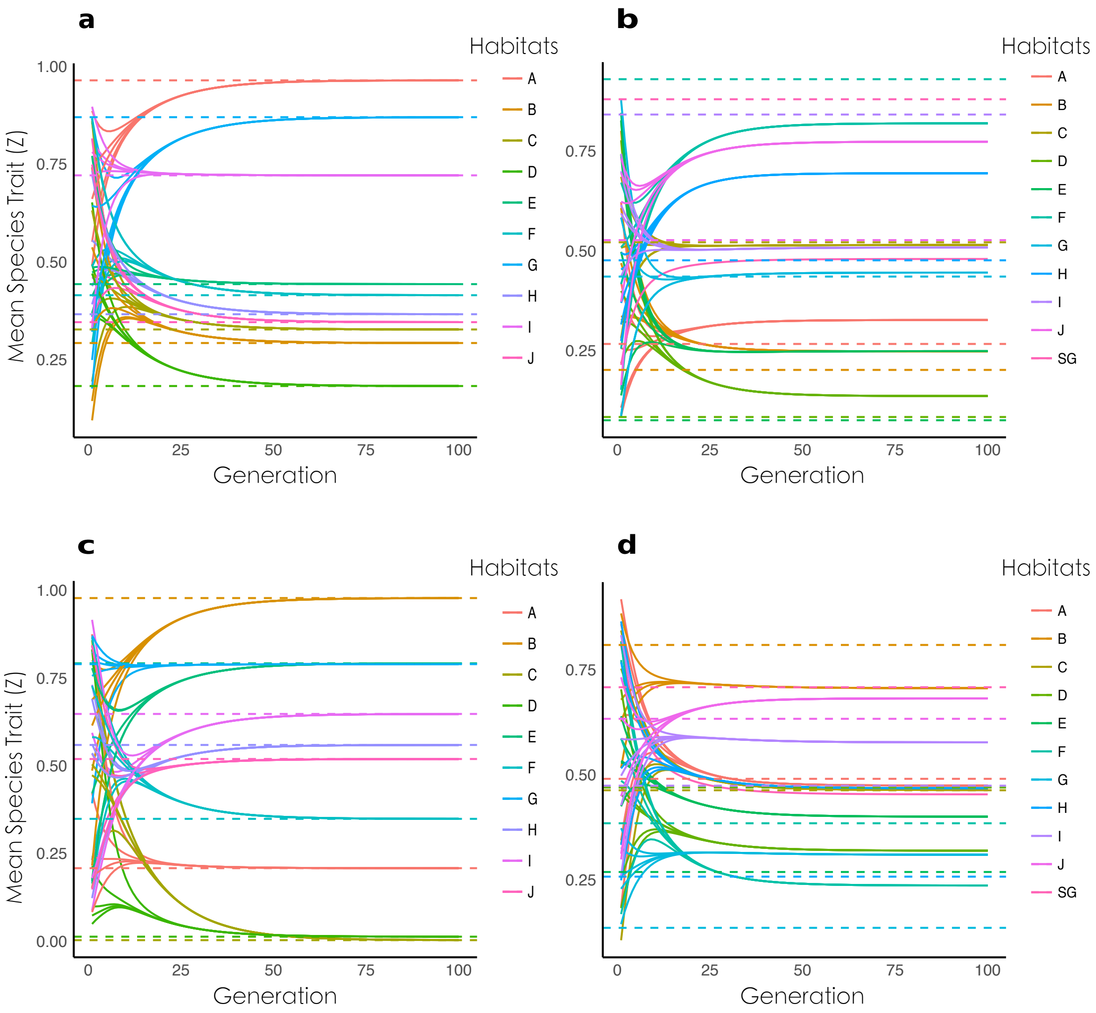

Figure S. 7: Sensitivity analyses of the scaling parameter α. Exploring evolutionary change in trait mean values under mimetic and habitat-based selection. Each habitat is represented by one color, dashed line is the habitat optima, each continuous line is one species and their color is the same as the habitat optima where they occur. In **a-b** α equals one **a)** species are loyal to their habit, occurring in only one habitat, in a *perfectly modular scenario* **b)** one species in the community is a supergeneralist. In **c-d** α equals four **c)** species are loyal to their habit, occurring in only one habitat, in a *perfectly modular scenario* **d)** one species in the community is a supergeneralist.

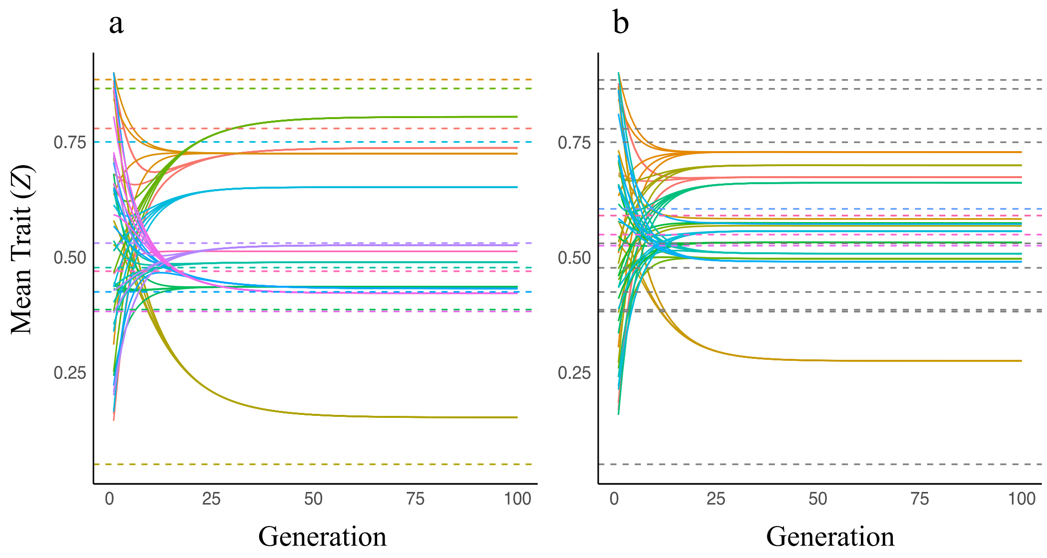

Figure S. 8: We performed simulation assuming supergeneralist species have a preference in one habitat. In this case, for each supergeneralist, we randomly chose a habitat and calculate the habitat optimum, in which this selected habitat would have triple weight than the others. We then used the weighted average to calculate the habitat optimum for the supergeneralists. Dashed line is the habitat optima, each continuous line is one species and their color is the same as the habitat optima where they occur. In **a** there is only one supergeneralist in the community. In **b** there are five supergeneralists in the community. Note that this simulation lead to similar results to our baseline simulations in the main text.

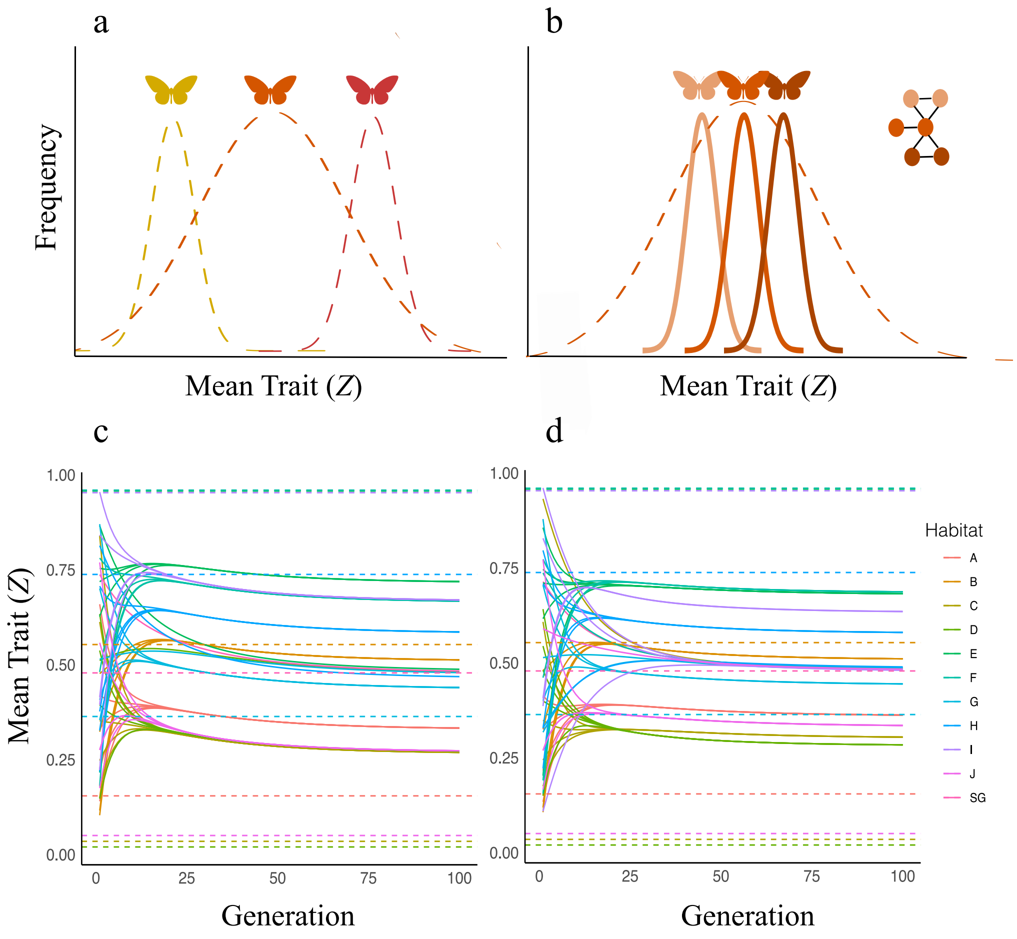

Figure S. 9: **A** and **b** are hypothetical curves describing the habitat trait optimum of species (dashed lines) and the expected frequency of trait (solid lines) in a scenario where there are two habits and species are habitat generalist occurring in more than one habitat. In **a,** although the habitat optimum for the generalist species is the mean value of the other two habitats, the slope of the curve is less steep, which means that there is a weaker penalty for species that are more distant to the optimum comparing with specialist species. In **b** we would expect the formation of three distinct rings, but they are very similar to each other and closer to the optimum of the generalist species. **C** and **d** show the sensitive analyses using a less steep curve in the adaptive landscape. For habitat supergeneralist species (SG) our scaling parameter $\varphi = 0.1$, whereas for specialist species our scaling parameter $\varphi = 0.25$. Each habitat is represented by one color, dashed line is the habitat optima, each continuous line is one species and their color is the same as the habitat optima where they occur. In **c** there are five supergeneralist species (SG) in the community and 45 habitat specialist species. In **d** there are ten supergeneralist species (SG) in the community and 40 habitat specialist species.

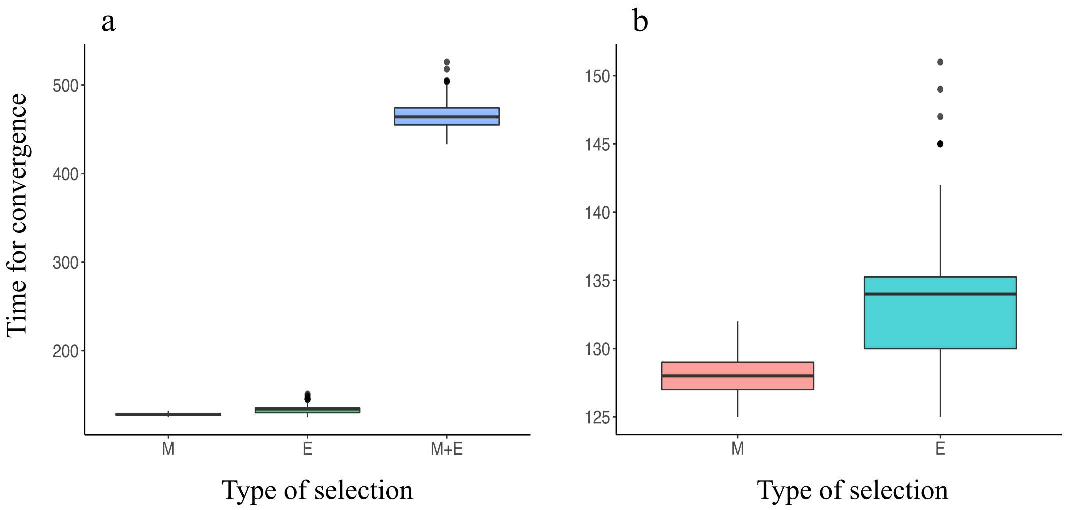

Figure S. 10: Both mimetic and habitat selection acting together lead to a longer time for convergence. **a)** Time for convergence assuming only mimetic (M), only habitat selection (E), and the interplay between the two (M+E) in the evolutionary dynamics; **b)** a focus on only mimetic (M) or only habitat selection (E) in the evolutionary dynamic.

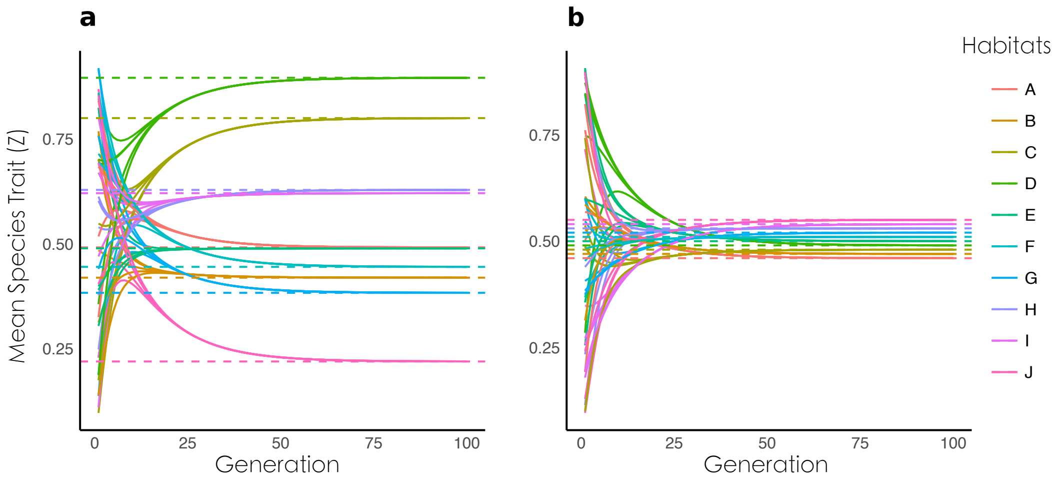

Figure S. 11: Exploring mimetic and habitat-based selection in an **a)** heterogeneous community, which has more distinct optimum values; **b)** homogenous community, which has very similar optimum values. Each habitat is represented by one color, dashed line is the habitat optima, each continuous line is one species and their color are the same as the habitat optima where they occur. All species are habitat specialists, occurring in only one habitat – *perfectly modular scenario*.

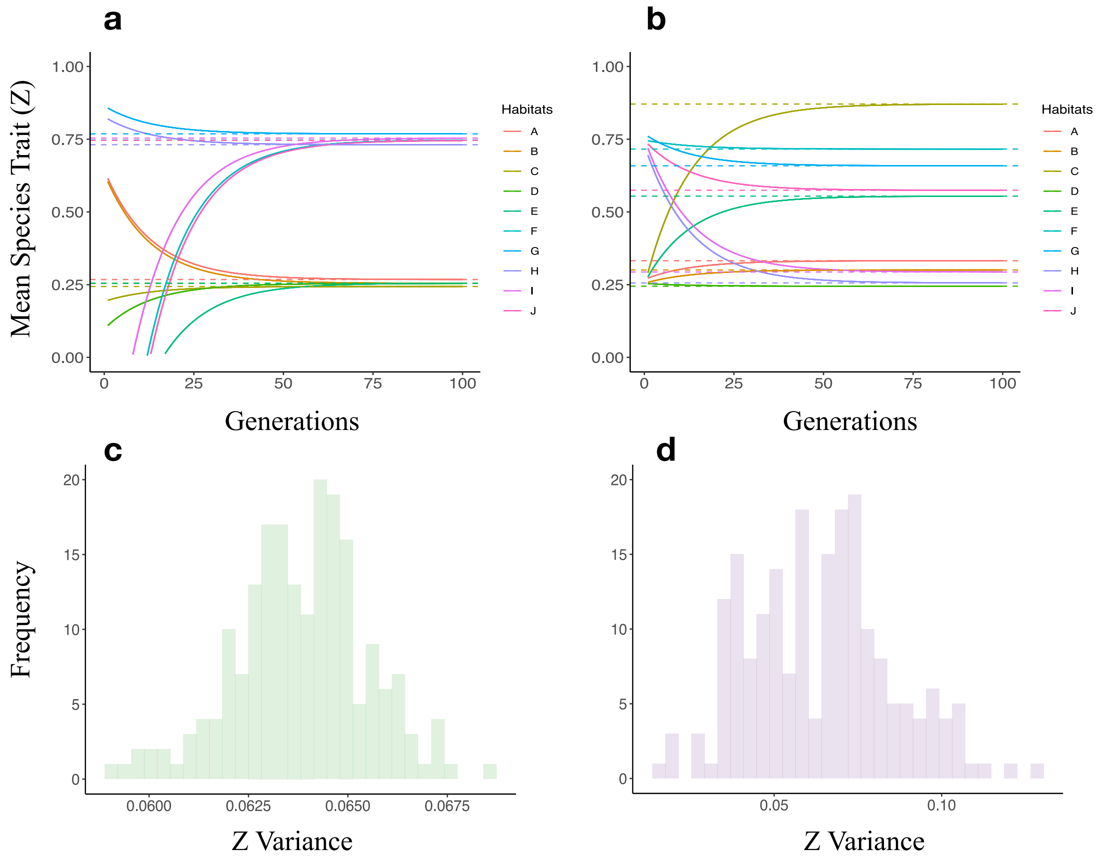

Figure S. 12: Exploring a scenario where thetas already favor the formation of groups. **a)** five θ_k were sampled from a normal distribution with mean= 0.25 and sd=0.01 and the others five$\theta_k$were sample from a normal distribution with mean=0.75 and sd=0.01. **b)** we generated a uniform distribution with the same theta variance (0.068) resulted in **a. C-d)** histograms describing the phenotypic variance across species when assuming **c)** the scenario of **a**; **d)** the scenario of **b.**

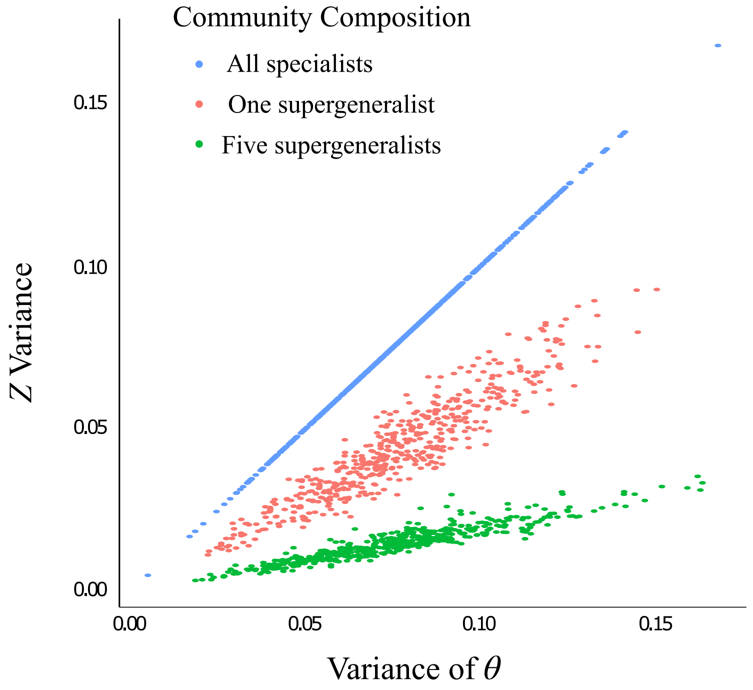

Figure S. 13: Increasing variance among habitats optima (θ) increases the variance among the mean trait value of species (Z) in the community. However, the trait variance is sensitive to community composition, in which having more supergeneralist species in the community decreases the variance among the mean trait value of species. Each color represents a different community composition, points are different simulation and the parameters were the same as in Figure 4.

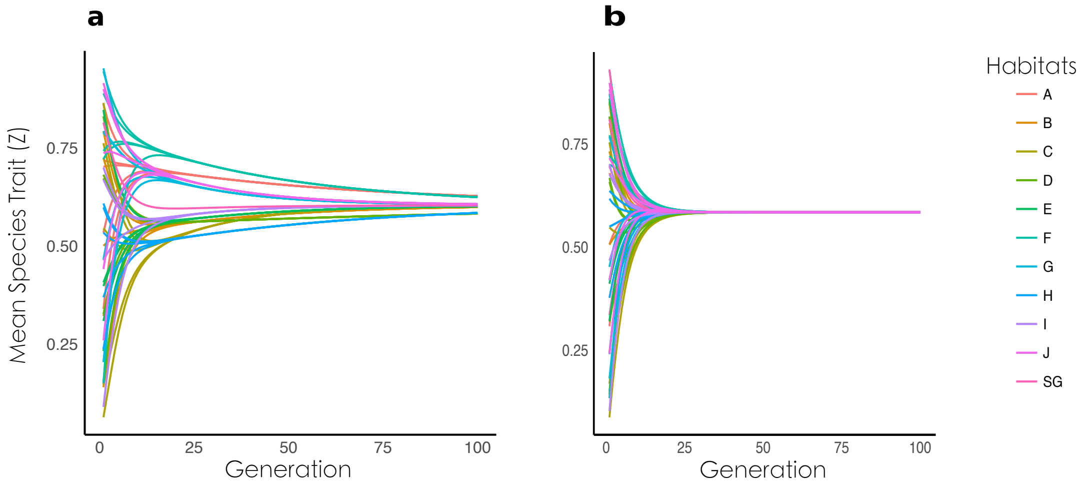

Figure S. 14: The effects of habitat-based selection (p = 0) and supergeneralist on the mean trait value (Z). Each habitat is represented by one color and continuous line is one species and their color are the same as the habitat optima where they occur. Starting from a *perfectly modular scenario* and allowing **a)** only one supergeneralist in the community; **b)** 10% of supergeneralist in the community

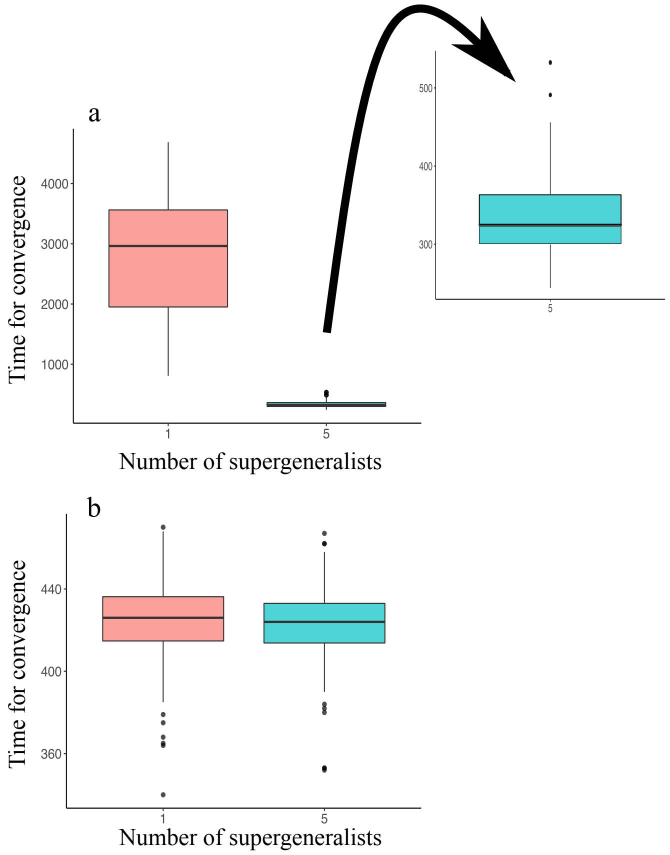

Figure S. 15: Exploring how the number of supergeneralist in the community affect the time for species to converge. **a)** when there is no habitat selection ($p = 0$); **b)** when both mimetic and habitat selection are acting in the evolutionary dynamic (p= 0.3).

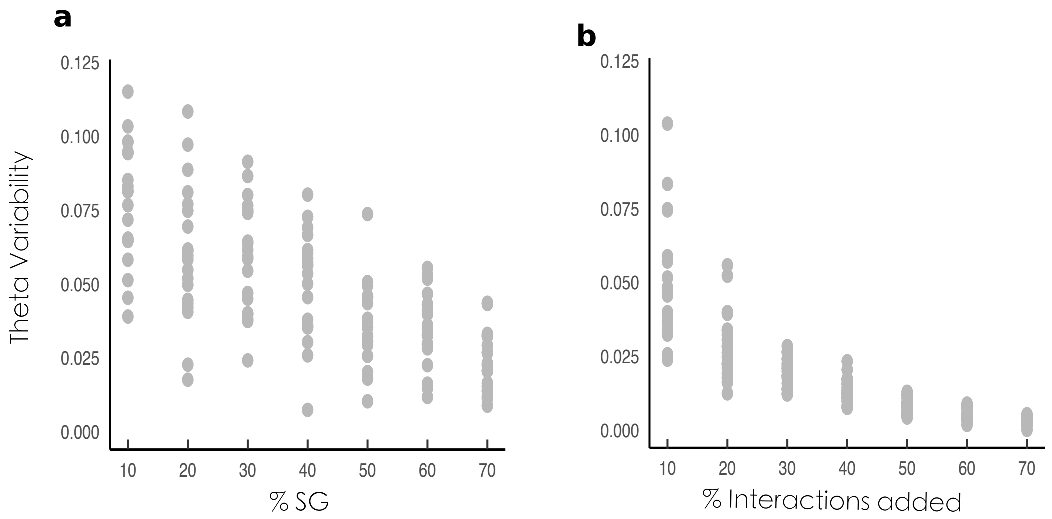

Figure S. 16: a) Variability of theta values among species in a community with different percentages of supergeneralist (SG). **b)** Variability of theta values among species with different percentages of occurrence added in a community. Each point corresponds to one simulation.

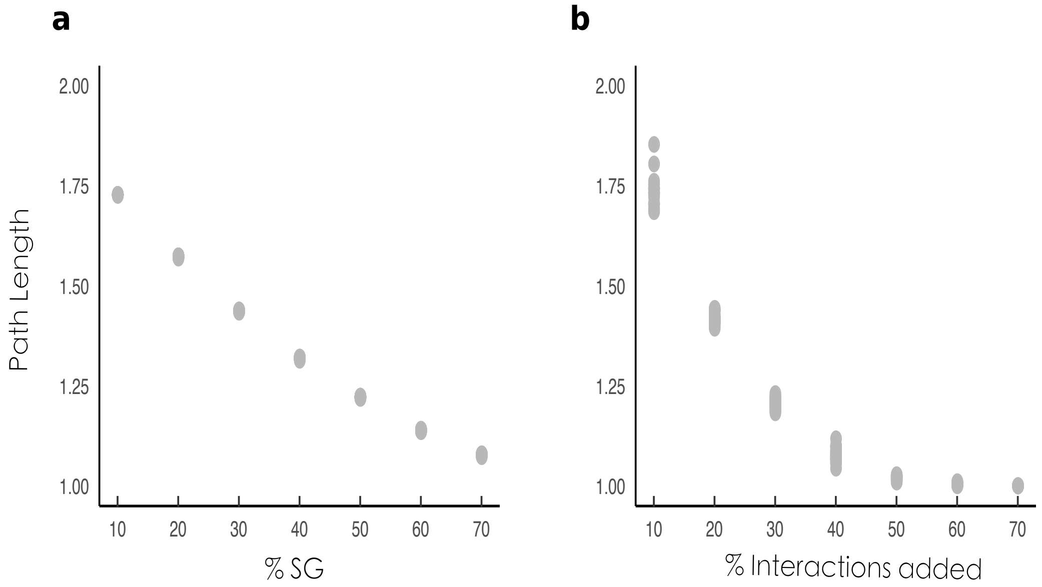

Figure S. 17: a) Path length between species in a community with different percentages of supergeneralist (SG). If species do not co-occur the path length between them tend to infinite. **b)** Path length between species with different percentages of occurrences in a community. Each point corresponds to one simulation.

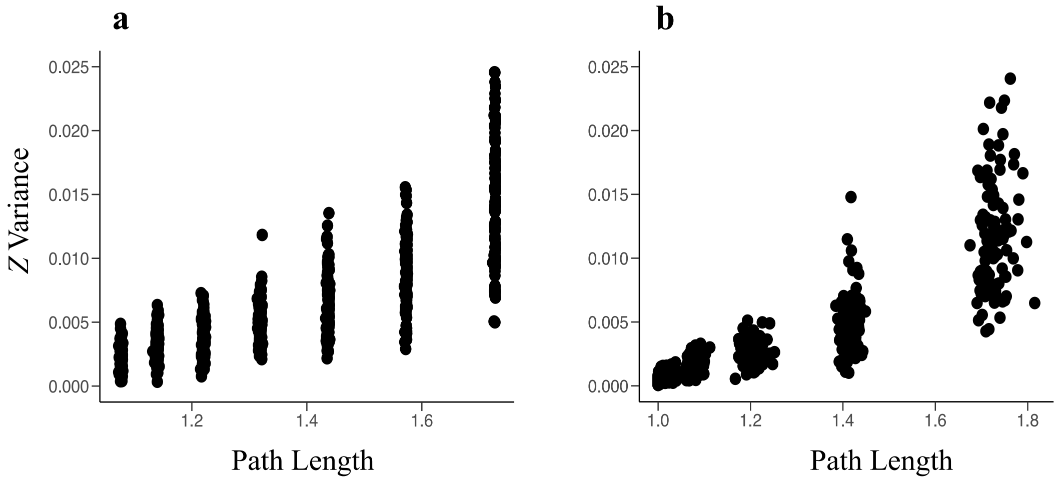

Figure S. 18: The effects of path length on the trait (Z) variance**. a)** Using the mean pairwise path length among species in a community with different percentages of supergeneralist (SG) – from 10% to 70%. If species do not co-occur the path length between them tend to infinite. **b)** Using the mean pairwise path length among species in a community with different percentages of new occurrences – from 10% to 70%. Each point corresponds to one simulation.

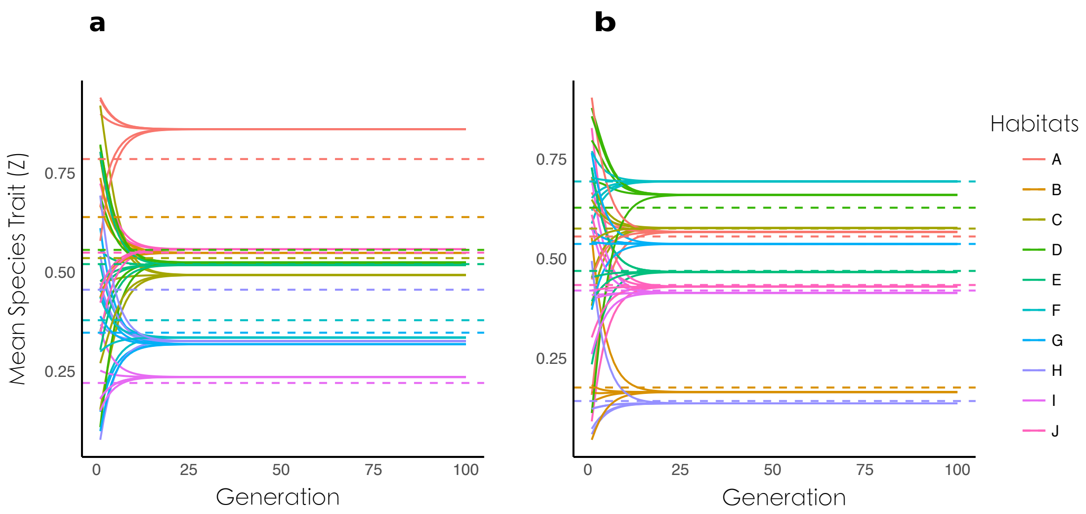

Figure S. 19: Exploring abundances and its effects on mimetic selection (p = 0). Each habitat is represented by one color, each continuous line is one species and their color are the same as the habitat optima where they occur. All species are habitat specialists, occurring in just one habitat. **a)** Dashed line is mean trait value at the first generations of species that occur in the same habitat **b)** Dashed line is the weighted mean trait value by abundance of species that occur in the same habitat

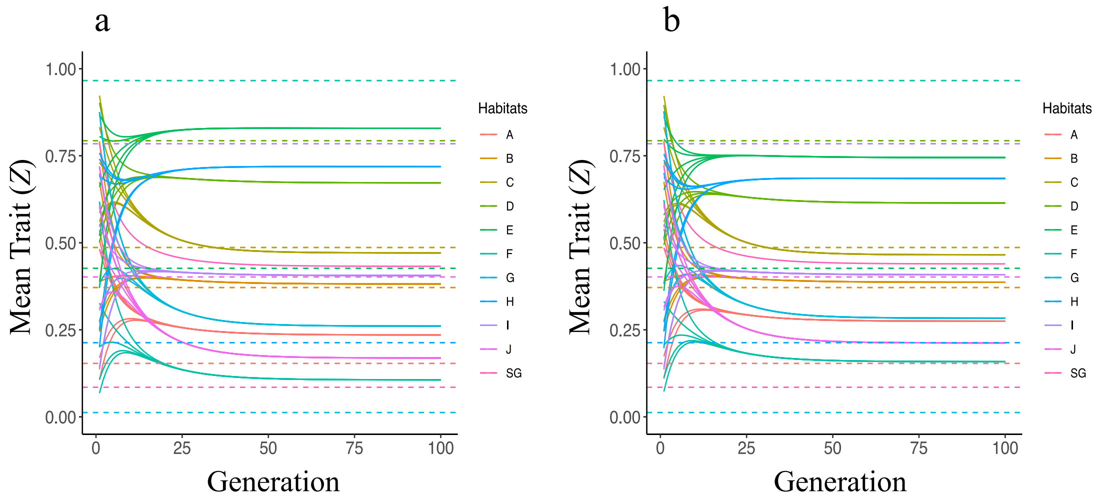

Figure S. 20: Exploring the effect of abundance of supergeneralist in habitat selection. We are comparing the effect of the habitat optimum of supergeneralist species weighted by their abundance with our default simulation. Each habitat is represented by one color, each continuous line is one species and their color are the same as the habitat optima where they occur. All species are habitat specialists, occurring in just one habitat. **a)** This is our default simulation, in which abundance only affects mimetic selection **b)** In this simulation habitat optimum is also affected by species abundance.
